# A Comparative Study of Transcranial Color-Coded Doppler (TCCD) and Transcranial Doppler (TCD) Ultrasonography Techniques in Assessing the Intracranial Cerebral Arteries Haemodynamics

**DOI:** 10.3390/diagnostics14040387

**Published:** 2024-02-10

**Authors:** Simon Takadiyi Gunda, Tsam Kit Veronica Ng, Tsz-Ying Liu, Ziman Chen, Xinyang Han, Xiangyan Chen, Marco Yiu-Chung Pang, Michael Tin-Cheung Ying

**Affiliations:** 1 Department of Health Technology and Informatics, The Hong Kong Polytechnic University, Hung Hom, Kowloon, Hong Kong SAR, China; simon.gunda@connect.polyu.hk (S.T.G.); tsam-kit-veronica.ng@connect.polyu.hk (T.K.V.N.); ty0119.liu@connect.polyu.hk (T.-Y.L.); ziman.chen@polyu.edu.hk (Z.C.); fiona.chen@polyu.edu.hk (X.C.); 2 Department of Radiography, National University of Science and Technology (NUST), Ascot Bulawayo P.O. Box AC 939, Zimbabwe; 3 Department of Rehabilitation Sciences, The Hong Kong Polytechnic University, Hung Hom, Kowloon, Hong Kong SAR, China; marco.pang@polyu.edu.hk

**Keywords:** cerebrovascular disease, transcranial Doppler ultrasonography (TCD), transcranial color-coded Doppler ultrasonography (TCCD), cerebral arteries, middle cerebral arteries, haemodynamics

## Abstract

Cerebrovascular disease (CVD) poses a major public health and socio-economic burden worldwide due to its high morbidity and mortality rates. Accurate assessment of cerebral arteries’ haemodynamic plays a crucial role in the diagnosis and treatment management of CVD. The study compared a non-imaging transcranial Doppler ultrasound (TCD) and transcranial color-coded Doppler ultrasound (with (cTCCD) and without (ncTCCD)) angle correction in quantifying middle cerebral arteries (MCAs) haemodynamic parameters. A cross-sectional study involving 50 healthy adults aged ≥ 18 years was conducted. The bilateral MCAs were insonated via three trans-temporal windows (TTWs—anterior, middle, and posterior) using TCD, cTCCD, and ncTCCD techniques. The MCA peak systolic velocity (PSV) and mean flow velocity (MFV) were recorded at proximal and distal imaging depths that could be visualised on TCCD with a detectable spectral waveform. A total of 152 measurements were recorded in 41 (82%) subjects with at least one-sided open TTW across the three techniques. The mean PSVs measured using TCD, ncTCCD, and cTCCD were 83 ± 18 cm/s, 81 ± 19 cm/s, and 93 ± 21 cm/s, respectively. There was no significant difference in PSV between TCD and ncTCCD (bias = 2 cm/s, *p* = 1.000), whereas cTCCD yielded a significantly higher PSV than TCD and ncTCCD (bias = −10 cm/s, *p* < 0.001; bias = −12 cm/s, *p* ≤ 0.001, respectively). The bias in MFV between TCD and ncTCCD techniques was (bias = −0.5 cm/s; *p* = 1.000), whereas cTCCD demonstrated a higher MFV compared to TCD and ncTCCD (bias = −8 cm/s, *p* < 0.001; bias = −8 cm/s, *p* ≤ 0.001, respectively). TCCD is a practically applicable imaging technique in assessing MCA blood flow velocities. cTCCD is more accurate and tends to give higher MCA blood flow velocities than non-imaging TCD and ncTCCD techniques. ncTCCD is comparable to non-imaging TCD and should be considered in clinical cases where using both TCD and TCCD measurements is needed.

## 1. Introduction

Cerebrovascular disease (CVD) poses a major public health and socio-economic burden worldwide due to its high morbidity and mortality rates [1,2]. Haemodynamic failure due to cerebral arteries stenosis and vulnerable atherosclerosis plaque rupture is the main mechanism of ischemic stroke [3,4,5]. However, intracranial cerebral artery stenosis (ICS) is deemed to account for most stroke cases (33% to 67%), especially in the Asian population [3]. The early identification and accurate classification of ICS is critical to inform treatment management and enhance prognostic outcomes in CVD patients as a selection of patients to undergo surgical revascularization and thrombolysis is informed via the degree of stenosis. ICS is directly assessed by establishing the percentage luminal reduction of intracranial cerebral arteries (ICAs) using the Warfarin–Aspirin symptomatic intracranial disease method (WASID) during angiographic techniques [6] or indirectly inferred from intracranial cerebral arteries’ (ICAs) haemodynamic parameters [7]. The accurate assessment of intracranial cerebral arteries’ (ICAs) haemodynamic, therefore, plays a crucial role in the diagnosis and treatment management of the cerebrovascular disease. Digital subtraction angiography (DSA) is the primary imaging modality for diagnosing ischemic cerebrovascular disease [5]. The imaging modality is, however, invasive and expensive. Other angiographic imaging modalities, such as computed tomography angiography (CTA) and magnetic resonance angiography (MRA), are also useful in the assessment of intracranial cerebral artery stenosis, but they are expensive and involve the administration of a contrast agent. The transcranial Doppler ultrasound (TCD) is a portable and non-invasive medical imaging technique that utilizes a low-frequency ultrasound (≤2 MHz) to assess the intracranial cerebral artery haemodynamic through thin-bone acoustic windows [8]. 

Clinically, there are two TCD techniques, i.e., the non-imaging TCD—commonly referred to as the transcranial Doppler ultrasound (TCD), and the imaging TCD—commonly known as the transcranial color-coded Doppler ultrasound (TCCD). Despite the non-imaging TCD being a common and validated technique in assessing ICA’s haemodynamic [9,10], it is still limited in routine clinical practice, as it cannot allow for the precise differentiation between individual vessels especially in the presence of anatomical variations [11]. The TCCD, on the other hand, combines B-mode and color Doppler ultrasound imaging, which facilitates the correct identification of ICAs through color-coding of the blood flow velocity [12]. This allows for an accurate placement of the Doppler sampling gate over the vessel for reliable measurement. The TCCD also offers the window for angle correction, enabling a more accurate measurement of blood flow velocities, whereas TCD assumes the insonation angle is less than 30 degrees [12,13]. Despite the inherent technical urge of TCCD over TCD, its application in ICA assessment has been limited, mainly due to its unavailability. Recent evidence points towards a surge in the acquisition and adoption of TCCD into clinical practice [14]. The study centre to which the non-imaging TCD system has been the sole TCD equipment available has also not been spared in this acquisition trend. It is worth noting that most previous studies have reported the ICA’s haemodynamic parameters based on TCD measurements with a paucity of data on TCCD [13,15]. Additionally, limited studies have assessed the inter-method agreement between TCD and TCCD to justify the possible inferences between the two techniques’ ICA haemodynamic measurements in follow-up cases or serial monitoring during or after treatment where using both TCD and TCCD measurements may be needed [16,17]. Moreover, the studies did not utilise the widely accepted and more accurate method of the Bland–Altman plot [18,19,20]. The middle cerebral arteries (MCAs) are the terminal end of the intracranial carotid artery and are responsible for 80% of the cerebral blood flow [21]. Furthermore, most stenotic lesions are reported to involve the MCAs [22]; hence, they are the commonly interrogated ICAs in the clinical workup of CVD. Traditionally, the MCA blood flow velocity has been widely used as a surrogate marker of cerebral blood flow [23,24]. 

Given the above background, the present study aimed to compare the non-imaging transcranial Doppler ultrasound (TCD) and transcranial color-coded Doppler ultrasound (with (cTCCD) and without (ncTCCD)) angle correction in quantifying the MCAs haemodynamic parameters. It is hypothesised that TCCD with angle correction will provide different haemodynamic measurements when compared to TCD and TCCD without angle correction. 

## 2. Materials and Methods

### 2.1. Compliance with Ethical Standards

This prospective cross-sectional study was approved by the Institutional Review Board of the Hong Kong Polytechnic University (HSEARS20220714001). All participants provided written informed consent prior to undertaking the ultrasound examinations. 

### 2.2. Study Population

A consecutive sample of 50 healthy volunteer adults of Chinese origin were enrolled via a public email call. The subjects’ inclusion criteria were healthy adults without a previous history of stroke or transient ischemic attack (TIA) and aged 18 years or older. The exclusion criteria were previous history of stroke or TIA, age < 18 years, and allergic to the ultrasound gel. The study sample size was informed by previous similar studies [25]. 

### 2.3. Data Collection Methods and Equipment

The Delica EMS-9PB TCD machine (Shenzhen Delica Medical Equipment Co., Ltd., Shenzhen, China), in conjunction with a 1.6 MHz pulsed wave transducer, was used for the non-imaging TCD assessment, while the Samsung RS85 ultrasound machine (Samsung Medison Co., Ltd., Seoul, Republic of Korea) equipped with a 1–5 MHz phased array transducer was used for TCCD assessment. The TCD and TCCD ultrasound scans of the MCAs for each subject were performed by a single sonographer with abundant experience in ultrasound scanning. Additionally, to cater for the possible physiological changes that may affect blood flow velocity over time, such as blood pressure and cognitive and motor activity [13], both examinations were performed on the same day, with subjects at rest.

The bilateral MCAs of the subjects were insonated via the trans-temporal window (TTW) at two standardised imaging depths (regions of interest, ROI): (1) proximal MCA segment at the bifurcation, and (2) distal portion of the MCA that could be visualised on the TCCD with a detectable spectral waveform across the TCD and TCCD techniques. The ROIs were identified by first scanning using the TCCD technique at three TTW locations (anterior, middle, and posterior), with the subject lying in a supine position as described in previous studies [26,27]. The TCCD protocol involved performing, firstly, an axial B-mode scan of the head, which was followed by color and spectral Doppler scans, respectively.

**Figure 1:**
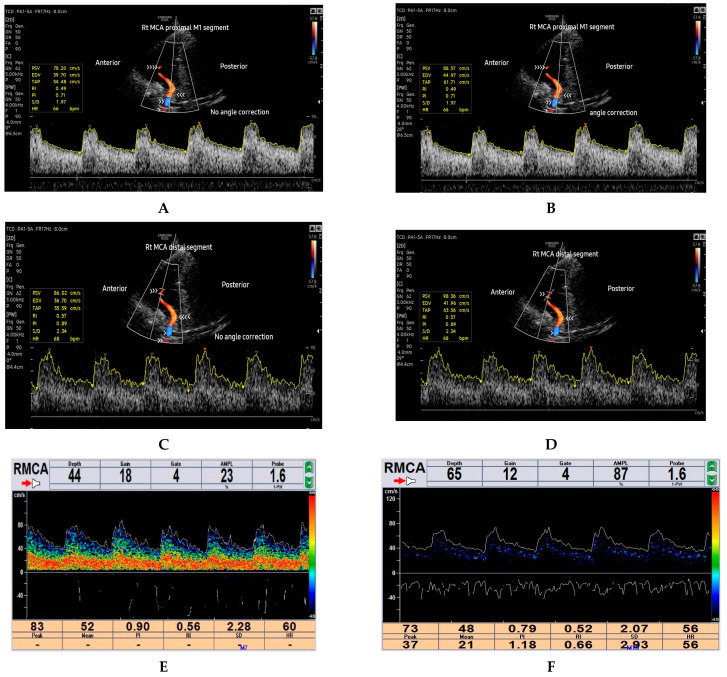
Images of a 40-year-old healthy subject showing the ROIs and the Doppler signal acquisition techniques: (**A**) ncTCCD measurement of PSV and TAP of proximal MCA (triple arrowheads), (**B**) cTCCD measurement of PSV and TAP of proximal MCA (triple arrowheads), (**C**) ncTCCD measurement of PSV and TAP of distal MCA (triple arrowheads), and (**D**) cTCCD measurement of PSV and TAP of distal MCA (double arrowheads). (**E**) TCD waveform showing measurement of PSV and MFV at a distal depth of MCA, (**F**) TCD waveform showing measurement of PSV and MFV at a proximal depth of MCA. The long vessel color-coded in red is the right MCA, whereas the ipsilateral anterior cerebral artery segment is color-coded in blue.

The ultrasound machine settings for the current study involved optimising the main parameters of B-mode ultrasound, such as power output, frequency, overall gain, time gain compensation (TGC), focusing, and depth. The optimisation was primarily achieved through the selection of a user-defined optimised preset under the TCD application available on the RS85 Samsung ultrasound machine (power output = 90%, frequency = general preset, dynamic range = 50, frame average = 8, scan area = 100%, focus = 1, and gain = 50). Minor adjustments to suit individual subjects were performed during the actual scanning. The depth would be increased to visualise the contra-lateral temporal skull bone and later adjusted to ensure that the ROI corresponding to ipsilateral proximal and distal MCA segments occupies at least 2/3 of the field of view (FOV) during velocity measurements, as shown in Figure 1A–D. A single focal zone was utilised and set at the level of the ROI to enhance lateral resolution. Additionally, color and spectral Doppler settings were optimised throughout the study. The color gain was optimised by first increasing the color gain until noise signals appeared, depicted as a vessel-bleed artifact outside the MCA blood vessel, then gradually decreased until the noise disappeared. The velocity scale or pulse repetitive frequency (PRF) was optimised by decreasing it until the aliasing artifact appeared and then increased to an optimum point until the aliasing artifacts disappeared. Furthermore, the sample volume was set at 4 mm for both the TCCD and TCD techniques. The typical B-mode, color, and spectral machine settings parameters used in this study are depicted in Supplementary Table S1.

In the B-mode axial scan plane, the mesencephalic brain stem structures were identified as hypoechoic, butterfly-shaped structures surrounded by a hyperechoic star-shaped basal cistern. Color Doppler imaging mode was then turned on to identify the ipsilateral MCA at the circle of Willis. Once the ipsilateral MCA was ascertained on color Doppler mode, a set of three consecutive measurements of the haemodynamic parameters (PSV and MFV) were performed at the two ROIs for the TCCD test methods (1) ncTCCD and (2) cTCCD using an automated spectral Doppler waveform analysis. The median of the three measurements was considered for data analysis. In ncTCCD, blood flow velocities were measured without applying angle correction. In cTCCD, the cursor for angle correction was applied and aligned parallel to the color Doppler blood flow direction before measuring the blood flow velocities, according to Polak et al. [28]. The same procedure was repeated by scanning the other side of the head. The MFV acquired in the TCCD techniques was represented by the time-averaged peak velocity (TAP). After completing the TCCD examinations, participants then proceeded to undertake the TCD examinations. During the TCD examination, the subjects were insonated via the same TTW location used in the TCCD examination using a 1.6 MHz hand-held probe. The uni-channel mode on the Delica EMS-9PB TCD machine was used, with the following typical parameters (probe frequency = 1.6 MHz, power output = 23.3, and gain—(8–18)). The sample volume depths were set at the two ROIs similar to those interrogated at TCCD, whereas a sample volume gate of 4 mm was similarly maintained across the techniques. An open TTW was defined as the ability to visualise the midbrain structures on the grayscale ultrasound image and the ipsilateral MCA. The ROIs and the Doppler signal acquisition techniques are shown in Figure 1A–F. 

### 2.4. Data Analysis

The IBM SPSS version 25 statistical package was used for data analysis. The ncTCCD and cTCCD MCA haemodynamic parameters (PSV and MFV) were compared with the TCD measurements for concordance. Both descriptive and inferential statistics were utilised. A one-way ANOVA and the non-parametric equivalent Kruskal–Wallis, followed by a Bonferroni post-hoc analysis, were used to compare the means of the PSV and MFV between the three techniques, respectively. The *p* < 0.05 was considered statistically significant. The mean difference (bias) and percentage differences in the MCA haemodynamic parameters between the three techniques were determined to represent the main outcome measures. The percentage difference of the MCA PSV among the three techniques, (1) TCD versus ncTCCD, (2) TCD versus cTCCD, and (3) ncTCCD versus cTCCD, was computed as the absolute value of the mean difference (bias) divided by the average of the MCA PSV of the two measurement techniques using (Equations (1)–(3)), respectively. The same comparisons were also applied to the MCA MFV measurement, and the percentage differences in the MFV across the three techniques were computed using Equations (4)–(6).

(1) $$D_{p}\left(TCD\;vs.\;ncTCCD,PSV\right)=\frac{\left|MD\right|}{\frac{TCD+ncTCCD}{2}}\;*\;100\%$$

(2) $$D_{p}\left(TCD\;vs.\;cTCCD,PSV\right)=\frac{\left|MD\right|}{\frac{TCD+cTCCD}{2}}\;*\;100\%$$

(3) $$D_{p}\left(ncTCCD\;vs.\;cTCCD,PSV\right)=\frac{\left|MD\right|}{\frac{ncTCCD+cTCCD}{2}}\;*\;100\%$$

(4) $$D_{p}\left(TCD\;vs.\;ncTCCD,MFV\right)=\frac{\left|MD\right|}{\frac{TCD+ncTCCD}{2}}\;*\;100\%$$

(5) $$D_{p}\left(TCD\;vs.\;cTCCD,MFV\right)=\frac{\left|MD\right|}{\frac{TCD+cTCCD}{2}}\;*\;100\%$$

(6) $$D_{p}\left(ncTCCD\;vs.\;cTCCD,MFV\right)=\frac{\left|MD\right|}{\frac{ncTCCD+cTCCD}{2}}\;*\;100\%$$

In Equation (1), $D_{p}\left(TCD\;vs.\;ncTCCD,PSV\right)$, represents the percentage difference (%) in peak systolic velocity (PSV) measurements between the two ultrasound techniques: the non-imaging transcranial Doppler ultrasound (TCD) and transcranial color-coded Doppler ultrasound, without angle correction (ncTCCD). Equations (2)–(6) follow similar formatting, whereas in Equations (4)–(6), the PSV is substituted by the mean flow velocity (MFV) as the measurement variable. 

*D_p_*—represents the percentage difference (%) and $\left|MD\right|$—represents the absolute value of the mean difference or bias of the variable measurement between two techniques. 

Additionally, Bland–Altman plots provided a visual assessment of the agreement between the techniques, TCD and TCCD (with and without angle correction), in measuring the MCA haemodynamic parameters. The differences between the TCD and TCCD (with and without angle correction) haemodynamic parameter values were plotted on the *Y*-axis against the average of the two measurements (*X*-axis). The limits of agreement (LOA) were calculated as the (bias ± 1.96 SD) and reflect the precision of the measurements. Two methods were deemed interchangeable when greater than 95% of the data points lie within the upper and lower LOA.

## 3. Results

### 3.1. Demographic Characteristics of the Study Participants

A total of 50 participants (17 men and 33 women; mean age, 49 ± 17 years; age range, 20 to 72 years) were enrolled between January and March 2023. All subjects underwent both TCD and TCCD examinations of MCAs. The demographic data of the study participants are presented in Table 1. The body mass index (BMI) of the subjects was (mean = 23 ± 4 kg/m^2^ and range = 17 to 32 kg/m^2^). Four subjects (8%) were underweight with a BMI < 19 kg/m^2^. Four subjects (8%) had a systolic blood pressure of over 140 mmHg, indicative of hypertension.

### 3.2. Trans-Temporal Window (TTW) Status in the Study Population

**Figure 2:**
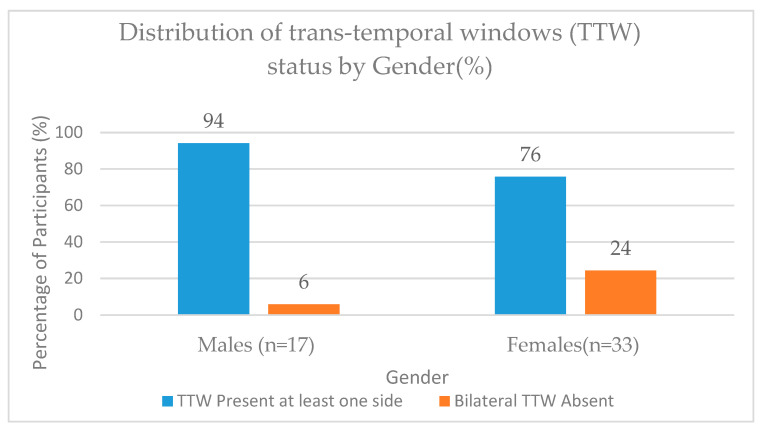
Histogram showing the trans-temporal window (TTW) status by gender.

A total of 41 subjects (82%) had at least a one-sided open TTW for assessing MCAs in TCD and TCCD (36 bilateral open TTW and 5 unilateral open TTW—3 on the left side and 2 on the right side), whereas 9 (18%) subjects had bilateral TTW failure to evaluate MCA. Finally, among the 50 subjects, a total of 77 MCAs were visualised, and haemodynamic parameters were interrogated on both TCD and TCCD techniques. In one subject, despite the presence of a bilateral open TTW, spectral Doppler signals could not be obtained at the same distal depths for the TCD and TCCD techniques on both sides of the head. A total of 152 MCA measurements were thus considered for blood velocity analysis (142 measurements from 36 subjects with a bilateral open TTW and 10 from 5 subjects with a unilateral TTW). A higher percentage of the presence of at least one side of an open TTW window was observed among male subjects in comparison to female subjects (94% versus 76%, respectively), and a corresponding higher percentage of TTW failure was observed in females compared to male counterparts (24% and 6%, respectively) (Figure 2). The observed gender-based differences in the TTW status were, however, statistically insignificant (χ^2^—test statistics = 2.562; df = 1; *p* = 0.109). The majority of the participants had a middle TTW, 27 (66%), and the remaining 14 (34%) had a posterior TTW. 

### 3.3. MCA Depths (Proximal and Distal) Interrogated and Doppler Angles

The mean proximal depth of the MCAs that could be visualised on TCCD was 59 ± 3 mm (range = 49–68 mm), while the mean distal depth was 44 ± 5 mm (range = 35–63 mm). The mean Doppler angle observed in the study was 24 ± 15 degrees. 

### 3.4. Comparison of All 152 MCA PSV Measurements across the Three Techniques (TCD, ncTCCD, and cTCCD)

**Figure 3:**
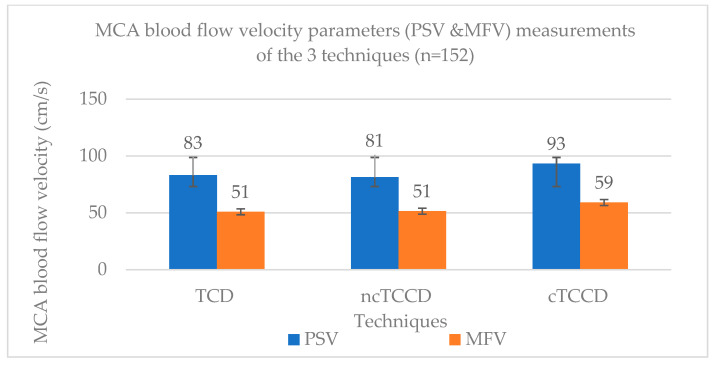
Histogram showing the mean MCA PSV and MFV (cm/s) across the three techniques.

**Figure 4:**
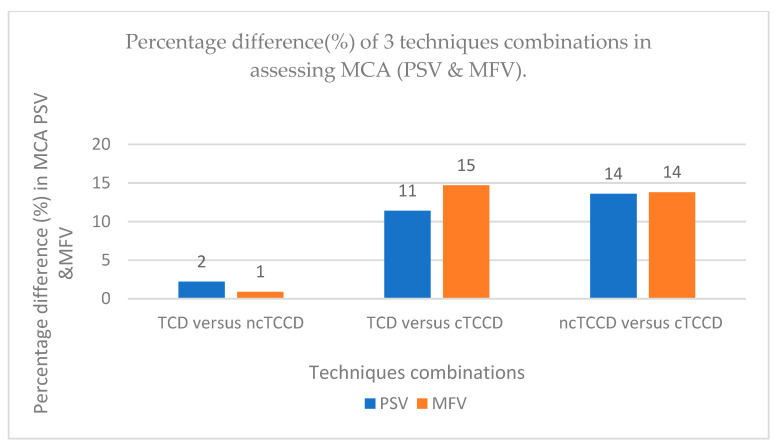
Histogram showing the percentage differences (%) in MCA PSV and MFV between the three technique combinations.

The mean MCA PSV measured using the TCD, ncTCCD, and cTCCD were 83 ± 18 cm/s, 81 ± 19 cm/s, and 93 ± 21 cm/s, respectively (Figure 3). One-way ANOVA results were significant (F- stats = 16.62; *p* < 0.001), and subsequent Bonferroni post hoc tests showed an insignificant difference in PSV measured between TCD and ncTCCD (t stat = 0.8245; *p* = 1.000), while a significant difference was observed between TCD and cTCCD (t = 4.53; *p* < 0.001). Additionally, MCA PSV measured using cTCCD was significantly higher than that measured using ncTCCD (t = 5.36; *p* < 0.001). The percentage differences in the PSV between TCD versus ncTCCD and TCD versus cTCCD techniques were 2% and 11%, respectively, while a 14% increase in the ncTCCD PSV was observed following angle correction (Figure 4). 

**Figure 5:**
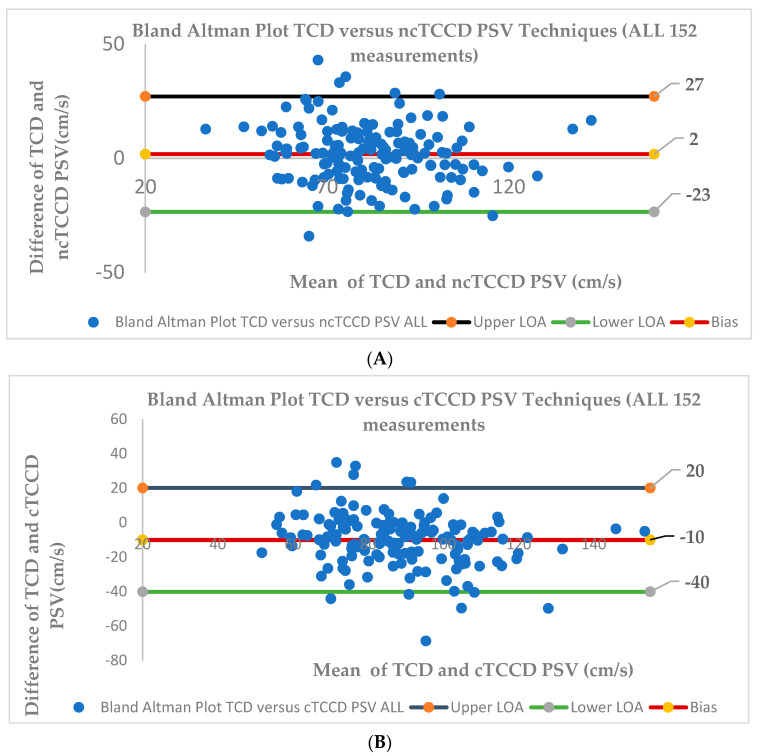
Bland–Altman plots for agreement between three techniques (TCD, ncTCCD, and cTCCD) in assessing MCA PSV (ALL 152 measurements). (**A**) TCD versus ncTCCD techniques, (**B**) TCD versus cTCCD techniques.

The Bland–Altman plot demonstrated a bias of 2 cm/s in PSV measurements between TCD and ncTCCD techniques (Figure 5A). The small positive value of the bias reflects that, on average, the ncTCCD technique minimally gives a lower PSV measurement in comparison to TCD. The LOA were 27 and −23 cm/s. A good agreement was observed between the TCD and ncTCCD techniques, as 95% of the data points lie within the LOA; hence, the two techniques are considered interchangeable in measuring the MCA PSV.

The bias in the PSV measurements between TCD and cTCCD techniques was −10 cm/s (Figure 5B). The relatively large negative value of the bias reflects that the cTCCD technique substantially yields a higher PSV measurement in comparison to the TCD technique. The limits of agreement were 20 and −40 cm/s. The Bland–Altman plot showed only 92% of the data points to lie within LOA; hence, the PSV measured using the cTCCD technique may not be interchangeable with that of TCD.

The red solid lines in A and B represent the mean of the difference (bias) in the MCA PSV measurement between the TCD and ncTCCD and TCD versus cTCCD techniques. The black and green lines represent the upper (ULA) and lower (LLA) limits of agreement, respectively. The ULA is given as the bias + 1.96 × standard deviation (SD), and the LLA is given as the bias − 1.96 × SD.

#### Comparison of the Proximal and Distal MCA PSV in Each of the Three Techniques

The mean MCA PSV measurements of the proximal and distal MCA segments of each of the three imaging techniques are shown in Table 2. In the three imaging techniques, the PSV measured at the proximal MCA was significantly higher than that measured at the distal MCA (*p* < 0.05). The mean proximal MCA PSV for the TCD, ncTCCD, and cTCCD techniques were 90 ± 15 cm/s, 88 ± 15 cm/s, and 100 ± 17 cm/s, respectively, while the corresponding mean distal MCA PSV were 76 ± 18 cm/s, 75 ± 21 cm/s, and 87 ± 22 cm/s, respectively. For both proximal and distal MCA, a significant difference in PSV between the TCD and cTCCD and between ncTCCD and cTCCD techniques was observed (*p* < 0.05). The MCA PSV between TCD and ncTCCD was, however, not significantly different from each other, regardless of the interrogated depth (*p* = 1.00). 

The Bland–Altman plots between TCD and ncTCCD in the measurement of proximal and distal MCA PSV are shown in Supplemental Figure S1A,B, respectively. A total of 95% of the proximal and 96% of the distal MCA measurement data points are within the LOA; hence, the two techniques are interchangeable regardless of the imaging depth. In the Bland–Altman plots between TCD and cTCCD, 94% and 93% of the data points are within the LOA for the proximal and distal MCA PSV measurements, respectively (Supplemental Figure S1C,D, respectively). 

### 3.5. Comparison of MCA MFV across the Three Techniques (TCD, ncTCCD, and cTCCD)

In the 152 measurements, the mean MCA MFV measured using TCD, ncTCCD, and cTCCD were 51 ± 11 cm/s, 51 ± 12 cm/s, and 59 ± 14 cm/s, respectively (Figure 3). The MFV measured using ncTCCD was not significantly different from that of TCD (stats = −0.529; *p* = 1.000), whereas there was a significant difference in the MFV between TCD versus cTCCD (stats = −5.142; *p* < 0.001) and ncTCCD versus cTCCD (stats = −4.613; *p* < 0.001) techniques. The percentage differences in assessing the MCA MFV between TCD versus ncTCCD, TCD versus cTCCD, and ncTCCD versus cTCCD were 1%, 15%, and 14%, respectively (Figure 4). 

**Figure 6:**
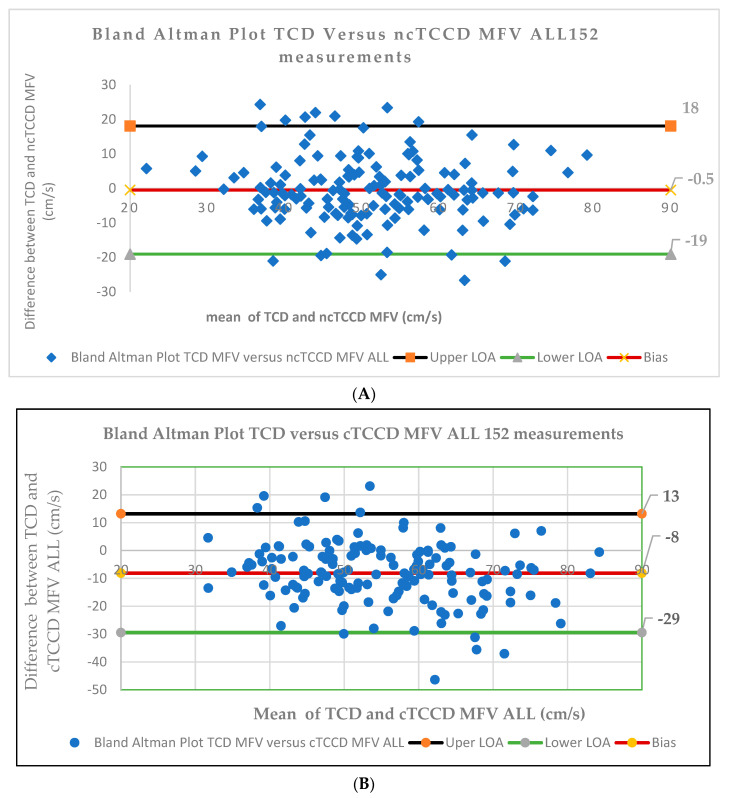
Bland–Altman plots for agreement between three techniques (TCD, ncTCCD, and cTCCD) in assessing MCA MFV (ALL 152 measurements). (**A**) TCD versus ncTCCD techniques, (**B**) TCD versus cTCCD techniques. The red solid lines in A and B represent the mean of the difference in the MCA MFV measurement between the TCD versus ncTCCD and TCD versus cTCCD techniques. The black and green lines represent the upper (ULA) and lower (LLA) limits of agreement, respectively. The ULA is calculated as the bias + 1.96 × standard deviation (SD), and the LLA is given as bias − 1.96 × SD. The limits of agreement (LOA) were calculated as the bias ± 1.96 SD and reflects the precision of the measurements.

The Bland–Altman plots for the comparison of the three techniques in assessing the MCA MFV measurements are shown in Figure 6. A bias of −0.5 was observed between the TCD and ncTCCD techniques, and the LOA were 18 and −19 cm/s, whereas 91% of the data points lay within the LOA. cTCCD exhibited higher MCA MFV when compared to the TCD technique, bias = −8 cm/s, and the LOA was 13 and −29 cm/s, whereas 93% of data points were within the LOA.

#### Comparison of the Proximal and Distal MCA MFV in Each of the Three Techniques

The mean proximal MCA MFV measured using TCD, ncTCCD, and cTCCD were 55 ± 9 cm/s, 56 ± 9 cm/s, and 63 ± 11 cm/s, respectively, and the corresponding distal MCA MFV were 46 ± 11 cm/s, 47 ± 13 cm/, and 55 ± 14 cm/s, respectively. The observed mean differences between TCD and ncTCCD techniques’ MFV measurements in both the proximal and distal MCA were not statistically significant (bias = −0.4 cm/s; *p* = 1.000, and bias = −0.6; *p* = 1.000, respectively). Contrarily, a significant difference was observed in the MFV measurements in both the proximal and distal MCA between TCD and cTCCD (bias = −8 cm/s; *p* < 0.001, and bias = −8; *p* < 0.001, respectively), and ncTCCD and cTCCD (bias = −8 cm/s; *p* < 0.001, and bias = −8; *p* < 0.001, respectively). 

The Bland–Altman plots for the comparisons of the three techniques in the assessment of the proximal and distal MCA MFV are shown in Supplemental Figure S2A–D. A marginal systematic bias was observed between the TCD and ncTCCD techniques proximal and distal MFV (−0.4 cm/s and −0.6 cm/s, respectively), while cTCCD yielded higher MFV when compared to TCD for both proximal and distal measurements (bias = −8 and 8 cm/s, respectively).

## 4. Discussion

This study compared TCD and TCCD with or without angle correction techniques in quantifying the MCA haemodynamic parameters and ascertaining whether the techniques are interchangeable. The present study demonstrated a significantly higher number (*n* = 41, 82%) of subjects with at least one side of an open TTW for TCD and TCCD examinations. In addition, the current study found that the majority of the open TTWs were middle TTW (χ^2^—test statistics = 4.122; *p* = 0.042). The higher prevalence of a middle TTW observed in the present study reaffirms the need to use the middle TTW as the initial location to focus the transducer when performing a TCD scan, as reported in a recent study by Chan et al. [26], as this has the potential of reducing the scan times. 

Based on the definition of TTW failure representing the number of MCA vessels that could not be visualised to allow for the interrogation of the haemodynamic parameters, a TTW failure rate of 23% was observed in this study, which is lower than that reported in previous studies (28.8–37%) [15,29,30]. As Asian subjects were used in the present and previous studies, the difference in the TTW failure rates between our study and previous studies could probably be attributed to the age difference. It has been reported that age is a significant factor in TTW failure rates, with higher failure rates observed in older adults than in younger populations due to increased temporal bone thickness associated with ageing [29,30,31]. In the present study, the mean age of the subjects was 49 ± 17 years, which was lower than that in previous studies (64.5 ± 13.2 to 65.1 ± 11.9 years) [15,29,30]. As anticipated, subjects with bilateral TTW failure were demonstrated to have a higher mean age of (58 ± 15 years) compared to the mean age of (46 ± 18 years) in those with a bilateral open TTW, thus further reaffirming that older age is linked to TTW failure. Previous studies have shown that bilateral TTW failure is more common in females [29,30,32]. In the present study, most subjects with bilateral TTW failure were females (*n* = 8, 89%). A further gender versus TTW status cross-tabulation, taking into account the significant gender inhomogeneity present in the current study, revealed a statistically insignificant difference in the TTW status across genders (Chi-squared statistics = 2.562; df = 1; *p* = 0.109). The significantly higher incidence of subjects with an open TTW, independent of gender, implies that TCCD can be a practical imaging tool to use in the Chinese population where intracranial artery stenosis is indicated to be the most common cause of ischemic stroke, accounting for 33% to 67% of stroke cases [3,33].

The current study established the MCA PSV and MFV values measured using TCD, ncTCCD, and cTCCD techniques among healthy Chinese adults. In the measurement of MCA MFV using the TCCD techniques, our results were lower than those reported in a study by Tsuchiya et al. [17], and a greater discrepancy was observed for the angle-correction technique (ncTCCD = 51 ± 12 cm/s vs. 55 ± 16 cm/s; cTCCD = 59 ± 14 cm/s vs. 87 ± 16 cm/s, respectively). The wider variations in the mean Doppler angles of (24 ± 15 vs. 49) degrees for our study and Tsuchiya et al. [17], respectively, explain the observed discrepancy in the MFV. 

The previous studies that compared TCD and TCCD techniques in assessing MCA haemodynamic parameters have not utilised the widely accepted and accurate method of the Bland–Altman plot to establish whether the techniques are interchangeable [16,34]. Based on the results of the Bland–Altman plots, the present study has established the existence of good agreement, hence interchangeability between the TCD and ncTCCD in assessing the MCA PSV. We observed that more than 95% of the data points lie within the limits of agreement, with no evidence of fixed bias. The mean difference of 1.83 cm/s and a *p*-value of 1.000 between the TCD and ncTCCD techniques implies that ncTCCD yields MCA PSV, which is lower but not statistically different from the TCD PSV. Contrarily, the cTCCD technique was demonstrated to yield significantly higher PSV values and was not interchangeable with the TCD technique in assessing the MCA PSV (<95% of data points were within limits of agreement; bias = −10 cm/s; *p* < 0.001). Similar to our study, previous studies have observed significant differences in MCA PSV derived between the TCD and cTCCD, with percentage differences ranging between 5 and 15% [34,35], whereas in the current study, a percentage difference of 11% was observed. 

Additionally, a marginal statistically insignificant bias of 0.48 cm/s between the TCD and ncTCCD MCA MFV measurements (*p* = 1.000) was noted in our study, although it could not reaffirm the interchangeability of the two techniques. In contrast, a significant difference in the MFV was observed between TCD versus cTCCD and ncTCCD versus cTCCD (*p* < 0.05). Our findings concur with those observed in Martin et al. [36]. TCD and ncTCCD apply a Doppler angle of zero degrees in computing the blood flow velocities; however, we observed a mean Doppler angle of 24 ± 15 degrees between MCA and ultrasound beam direction in the current study. Hence, this probably explains the significantly higher velocities derived using the cTCCD technique in comparison to both TCD and ncTCCD in the current study. cTCCD may, therefore, provide more accurate measurements of the MCA blood flow velocities, and where comparisons to previous TCD and TCCD without angle correction are required, caution should be taken to consider the observed biases between the techniques. The Doppler angle corrections reported in our study were less than the mean insonation angles reported in previous studies by Bartels and Flugel [34] and Eicke [37]. Furthermore, in previous studies, the Doppler measurements were mainly performed along the horizontal section of the MCA M1 segment, whereas in the current study, we interrogated the distal and proximal portions of the MCA, which did not necessarily correspond to the MCA M1 horizontal portion. The variations in the ROI interrogated between the current and previous studies may further explain the differences between the studies. 

In the current study, the distal and proximal MCA reference depths that could be interrogated using TCCD based on the direct visualisation of the color-coded MCA among healthy Chinese adults were reported for the first time. Previous studies that have compared the two methods, TCD and TCCD, interrogated the blood flow velocities at predetermined fixed depths without taking into consideration the possible anatomical variations in the subjects’ MCAs. As one’s proximal depth may actually correspond to another subject’s distal segment depth, using the fixed-depth approach may result in measuring flow velocities derived at anatomically different positions of the MCAs across the participants. The current study provided a more anatomically based standardised methodological approach by focusing on the proximal and distal reference portions of the MCAs. In one study, a cut-off depth ranging between 45 mm and 60 mm was used as the criteria to identify the MCA blood flow [29], whereas the current study reaffirmed these cut-off values when the mean of the distal and proximal depths were used (44 mm and 59 mm), respectively. However, the depth at which MCA signals could be observed ranged between 35 mm and 68 mm in the present study. Furthermore, the proximal and distal MCA haemodynamic parameters in all three techniques were observed to be significantly different from each other, with higher velocities recorded proximally than the distal MCA portion. The observed high proximal MCA velocities compared to distal MCA may be explained by anticipated high blood pressure at the proximal MCA segment, close to bifurcation, which in turn is reported to be associated with an increase in MCA velocities [24]. Due to the significant difference in the MCAs’ haemodynamic parameters measurements between the proximal and distal portions, it is imperative for clinicians to state the interrogation depth in reporting the MCAs’ haemodynamic measurements regardless of the TCD/TCCD technique employed. 

The use of a single sonographer to perform both the TCD and TCCD examinations has the potential to introduce some recall bias. To mitigate this possible limitation, a set of three consecutive measurements was taken, and the median values were considered. Additionally, the present study findings are applicable to the Chinese population as this study recruited Chinese subjects. Further studies are needed to verify the applications of TCCD in other ethnic populations. However, as a strength, the imaging depth was successfully standardised between the techniques to ensure that velocities from the same blood flow samples are interrogated and compared between the TCD and TCCD techniques. Furthermore, the TCD and TCCD examinations were both performed on the same day and time to cater to any possible physiological changes that may occur over time.

## 5. Conclusions

This study validated TCCD as a practically applicable imaging technique. TCCD with angle correction is a more accurate technique that tends to yield higher MCA blood flow velocities than non-imaging TCD and ncTCCD. Furthermore, ncTCCD is comparable to non-imaging TCD and should be considered in clinical cases where using both TCD and TCCD measurements are needed, such as in follow-up cases or serial monitoring during or after treatment, where patient baseline results are undertaken using another method. Finally, the study reaffirmed the importance of reporting the interrogation depth in MCA haemodynamics assessment as significant differences between proximal and distal blood flow velocities exist. 

## Figures and Tables

**Table 1:** Demographic characteristics of the study participants (*n* = 50).

	Age (years)	Weight (kg)	Height (cm)	BMI (kg/m^2)^	B.Ps (mmHg)	BPd (mmHg)	HR (BPM)
Mean	49	61	163	23	118	79	76
Standard deviation (SD)	17	12	8	4	14	9	12
Minimum	20	44	148	17	89	60	44
Median	57	60	164	22	116	82	75
Maximum	72	95	178	32	154	94	119
Normality test (KS)	0.223	0.144	0.096	0.116	0.114	0.126	0.128
Normality test *p*-value	0.014	>0.100	>0.100	>0.100	>0.100	>0.100	>0.100

BMI, body mass index; B.Ps, systolic blood pressure; BPd, diastolic blood pressure; HR, heart rate; BPM, beats per minute. The units of the demographic variables are written in brackets.

**Table 2:** Descriptive statistics of the proximal and distal MCA PSV measurements in the three techniques.

	PSV (cm/s)			
	Minimum	Maximum	Mean	Std. Deviation
Proximal MCA (*n* = 77) *				
TCD	66	151	90 ^#^	15
ncTCCD	55	134	88 ^†^	15
cTCCD	60	156	100 ^#†^	17
Distal MCA (*n* = 75) *				
TCD	43	144	76 ^#^	18
ncTCCD	30	131	75 ^†^	21
cTCCD	52	153	87 ^#†^	22

* Significant difference (*p* < 0.05) between proximal and distal MCA in the three techniques; ^#^ significant difference (*p* < 0.05) between TCD and cTCCD; ^†^ significant difference (*p* < 0.05) between ncTCCD and cTCCD.

## Data Availability

The datasets of this study are presented in the article/supplementary material. Any further inquiry can be directed to the corresponding author/s.

## Supplementary Materials

The following supporting information can be downloaded at: https://www.mdpi.com/article/10.3390/diagnostics14040387/s1, Figure S1: (A–D) Bland–Altman plots for agreement between the techniques, TCD versus ncTCCD, and TCD versus cTCCD in assessing the proximal and distal MCA PSV; Figure S2: (A–D) Bland–Altman plots for agreement between the techniques, TCD versus ncTCCD, and TCD versus cTCCD in assessing the proximal and distal MCA MFV; Table S1: RS85 Samsung Ultrasound machine Protocol settings.
