# Supplementary material for: A Comparative Study of Transcranial Color-Coded Doppler (TCCD) and Transcranial Doppler (TCD) Ultrasonography Techniques in Assessing the Intracranial Cerebral Arteries Haemodynamics
Source: Diagnostics (Basel). 2024 Feb 10;14(4):387. doi: 10.3390/diagnostics14040387 (PMC10887923; doi:10.3390/diagnostics14040387)
Supplement: Supplementary file 1 [file diagnostics-14-00387-s001.zip › diagnostics-2835446-supplementary.pdf]

Supplementary Figures.

Supplementary Figure S1. Bland-Altman plots for agreement between the techniques, TCD versus ncTCCD, and TCD versus cTCCD in assessing the proximal and distal MCA PSV.

(A). TCD versus ncTCCD (77 proximal MCA PSV measurements),

(B). TCD versus ncTCCD (75 distal MCA PSV measurements)

(C). TCD versus cTCCD (77 Proximal MCA PSV measurements),

(D) TCD versus cTCCD (75 distal MCA PSV measurements)

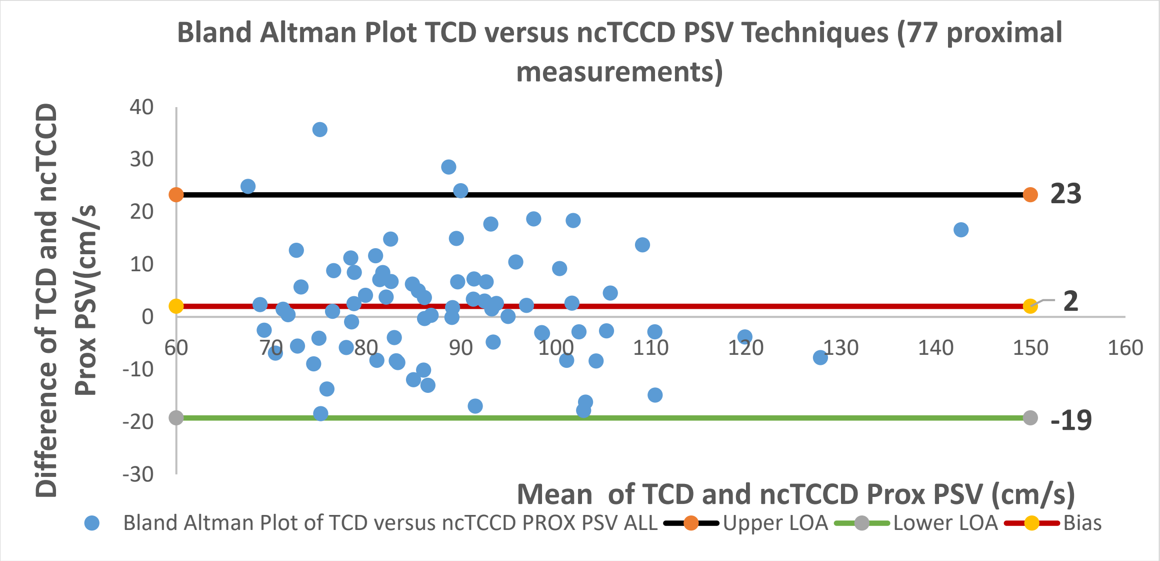

Figure S1A. Bland Altman plot TCD versus ncTCCD MCA PSV (proximal measurements).

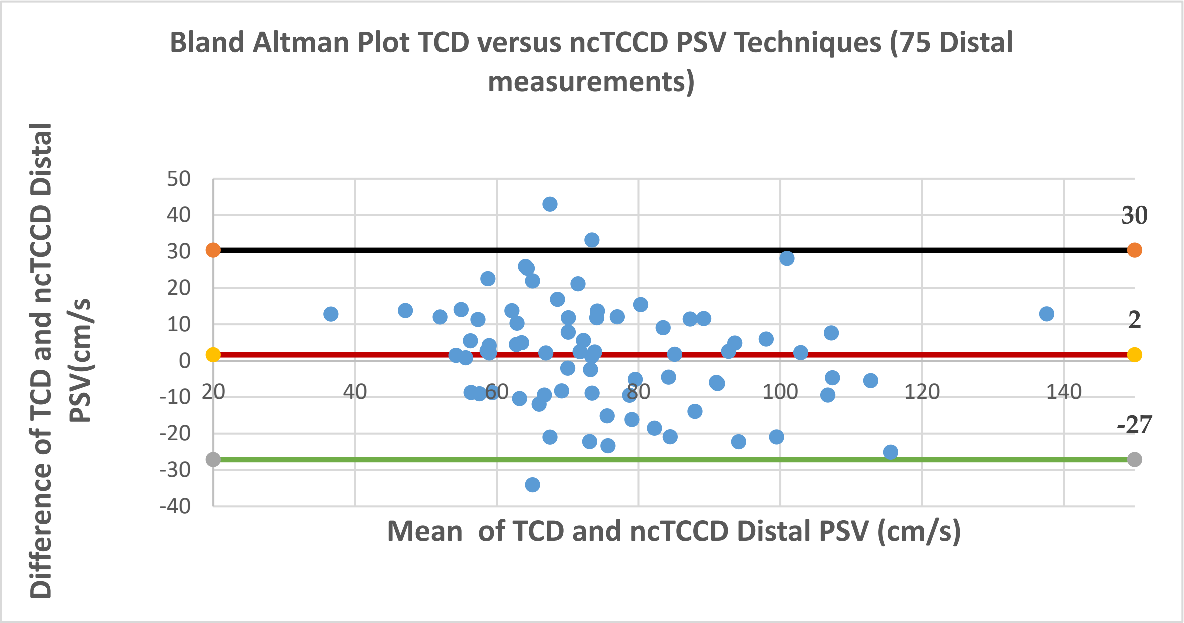

Figure S1B. Bland Altman plot TCD versus ncTCCD MCA PSV (Distal measurements).

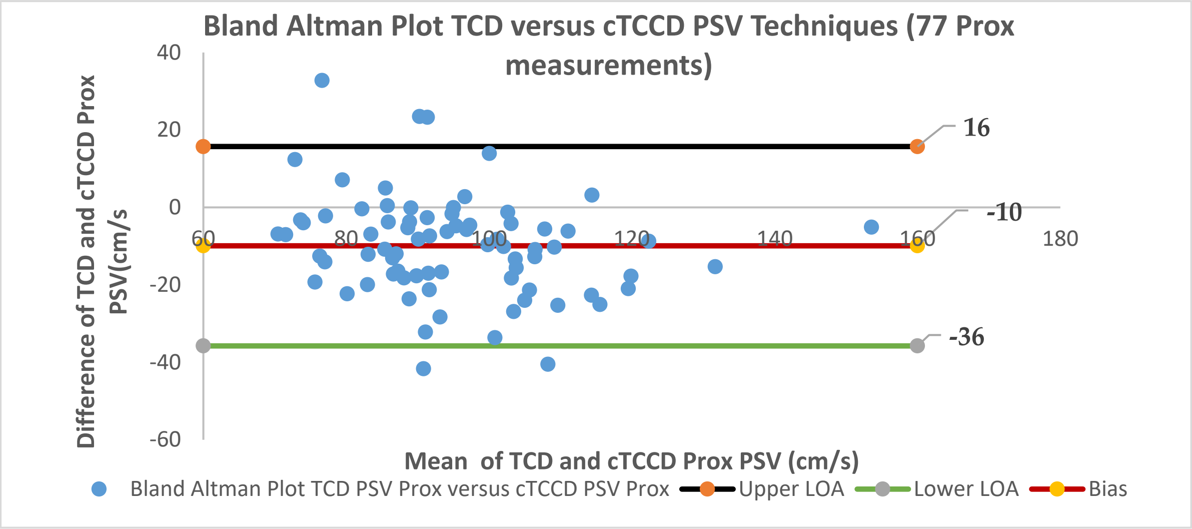

Figure S1C. Bland Altman plot TCD versus cTCCD MCA PSV (Proximal measurements)

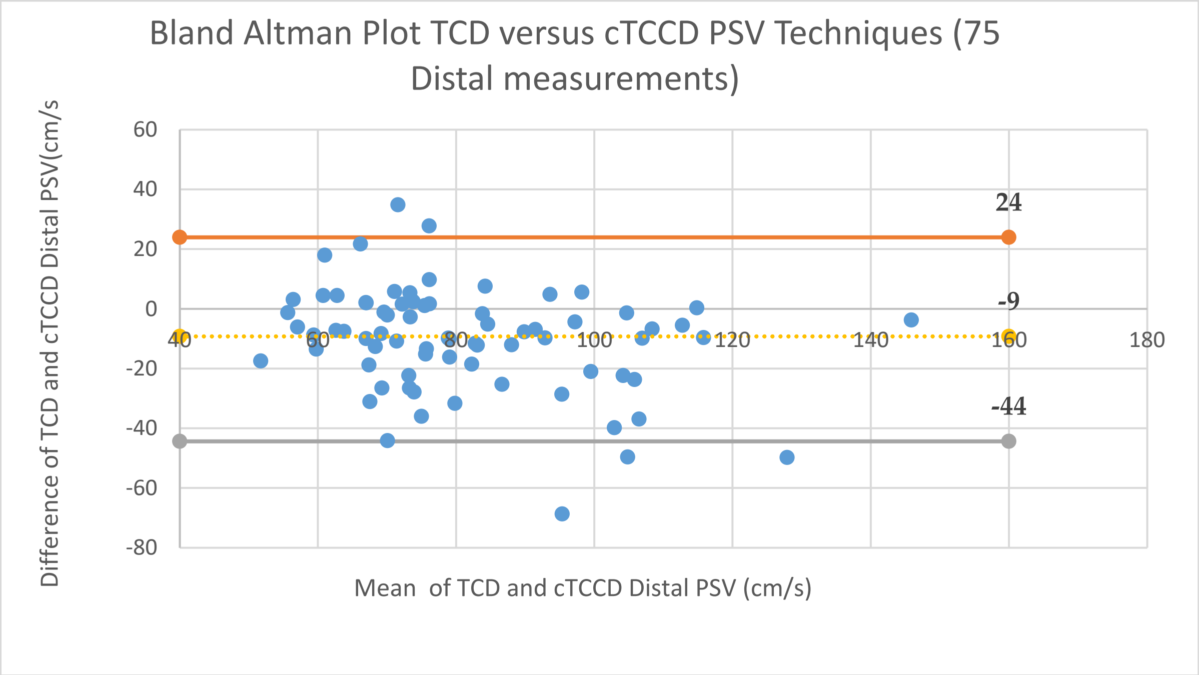

Figure S1D. Bland Altman plot TCD versus cTCCD MCA PSV (Distal measurements)

Supplementary Figure S2. Bland-Altman plots for agreement between the techniques, TCD versus ncTCCD, and TCD versus cTCCD in assessing the proximal and distal MCA MFV.

(A). TCD versus ncTCCD (77 proximal MCA MFV measurements),

(B). TCD versus ncTCCD (75 distal MCA MFV measurements)

(C). TCD versus cTCCD (77 Proximal MCA MFV measurements),

(D) TCD versus cTCCD (75 distal MCA MFV measurements)

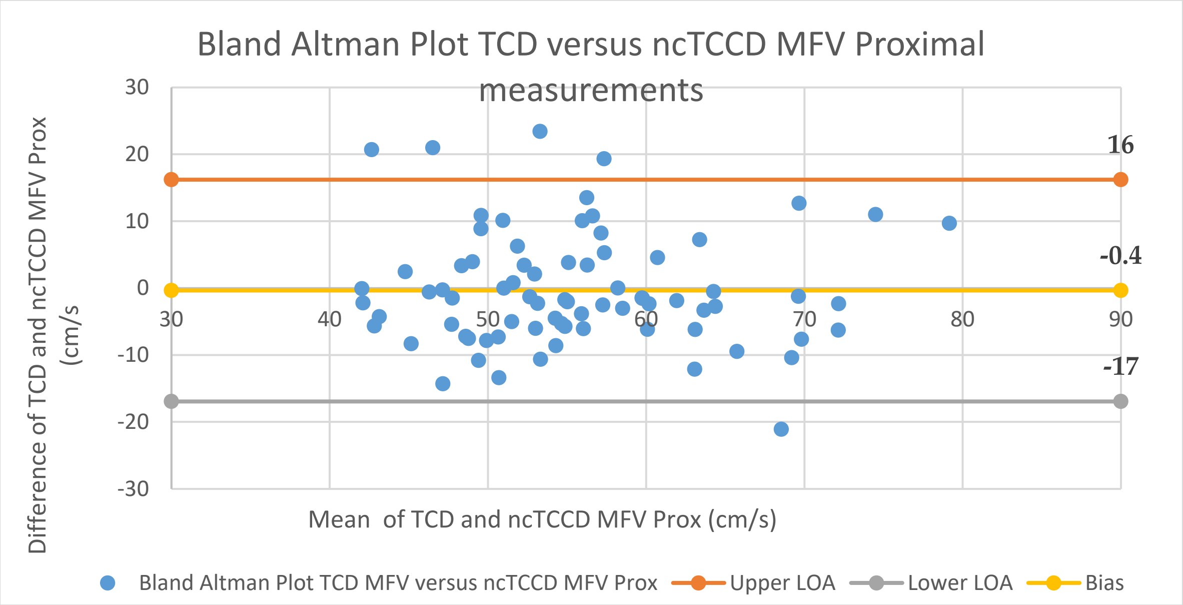

Figure S2A. Bland Altman plot TCD versus ncTCCD MCA MFV (77 Proximal measurements).

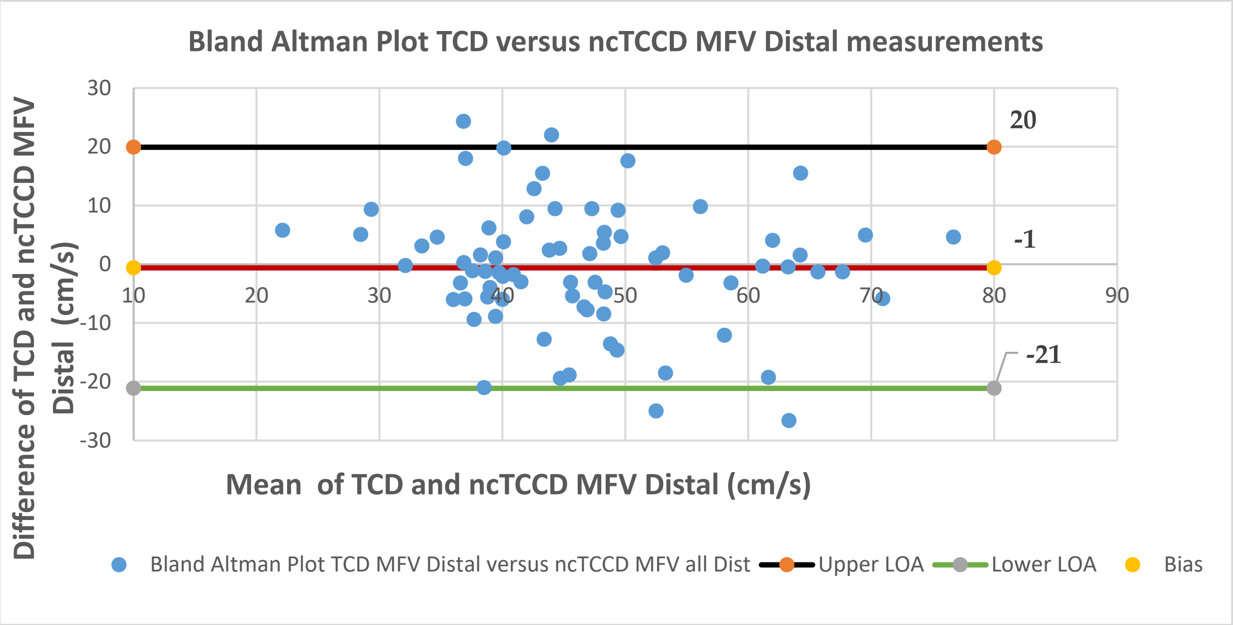

Figure S2B. Bland Altman plot TCD versus ncTCCD MCA MFV (75 Distal measurements).

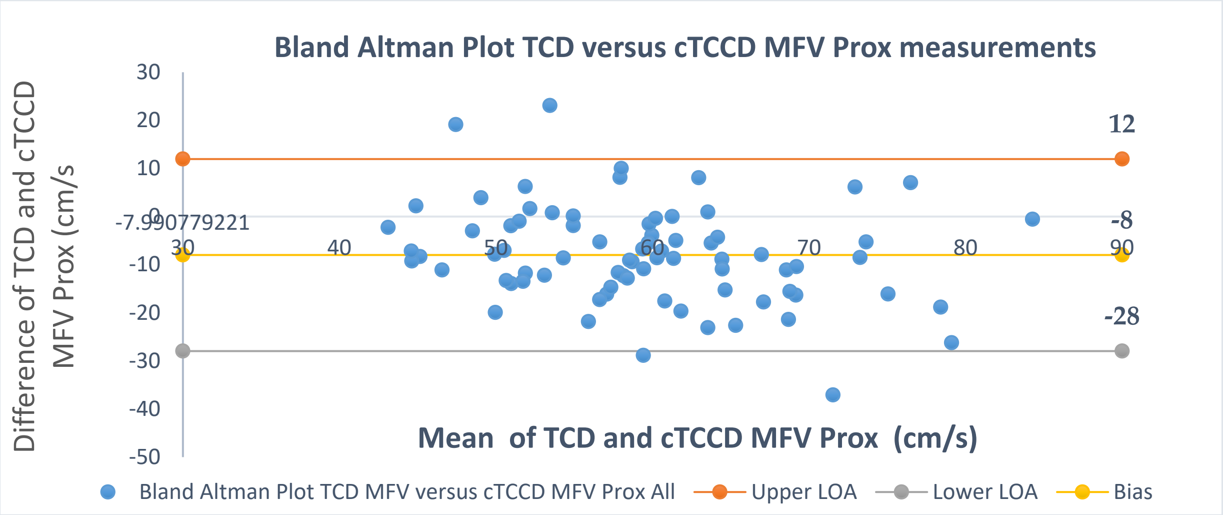

Figure S2C. Bland Altman plot TCD versus cTCCD MCA MFV (77 Proximal measurements).

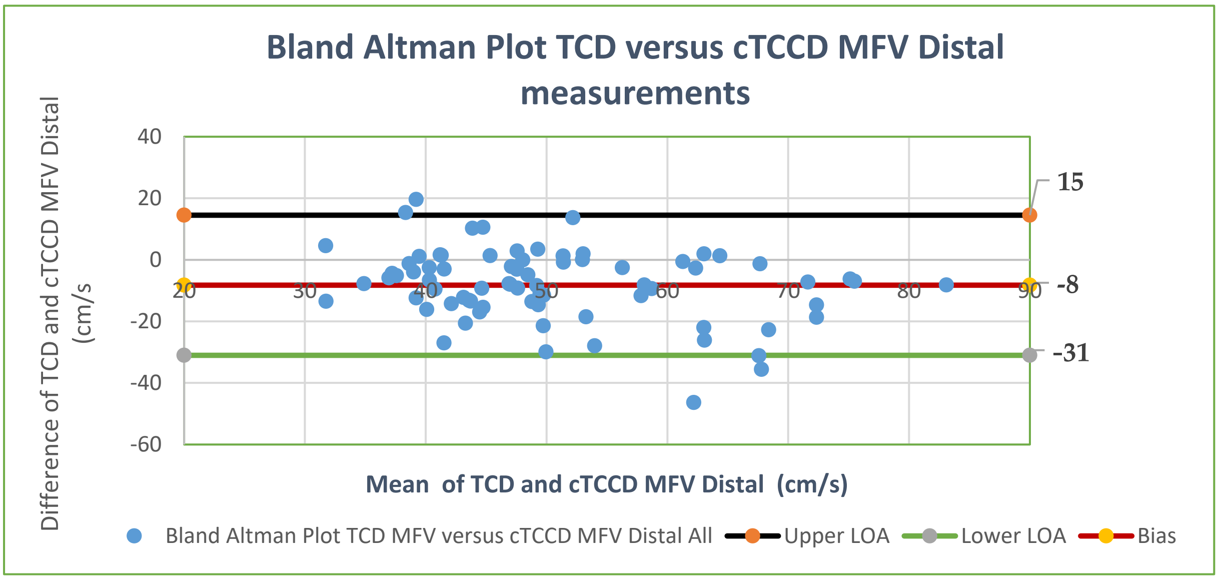

Figure S2D. Bland Altman plot TCD versus cTCCD MCA MFV (75 Distal measurements)

Supplementary Table S1: RS85 Samsung Ultrasound machine Protocol settings.

**2D mode**	**Color Doppler mode**	**Spectral Doppler mode**
Power=90	Power=90	Power=90
Probe Frequency-General Preset (1-5Mhz)	Gain-variable (start with high gain to optimum	Frequency=General preset
Dynamic Range=50	Persistence-	Gain=50
Gray map=7	Color box- adjusted to cover ROI.	Wall filter=1
Frame Average=8		Scale/PRF=variable (optimized to avoid aliasing artifact,& reduced in low velocity settings)
Scan area=100%		Sample volume size=4mm
Focus number=1		
Depth- adjusted until ROI occupy ~2/3 FOV.		
